# Novel *N*-Alkyl 3-(3-Benzyloxyquinoxalin-2-yl) Propanamides as Antiproliferative Agents: Design, Synthesis, In Vitro Testing, and In Silico Mechanistic Study

**DOI:** 10.3390/molecules30143025

**Published:** 2025-07-18

**Authors:** Samar A. Abubshait

**Affiliations:** 1 Department of Chemistry, College of Science, Imam Abdulrahman Bin Faisal University, P.O. Box 1982, Dammam 31441, Saudi Arabia; sabubshait@iau.edu.sa; Tel.: +966-504988846; 2 Basic and Applied Scientific Research Center, Imam Abdulrahman Bin Faisal University, P.O. Box 1982, Dammam 31441, Saudi Arabia

**Keywords:** benzyloxyquinoxaline, azide coupling, primary and secondary amines, anticancer

## Abstract

A series of eleven new *N*-alkyl 3-(3-benzyloxyquinoxalin-2-yl) propanamides were prepared based on the azide coupling of 3-(3-benzyloxyquinoxalin-2-yl) propanhydrazide with a variety of primary and secondary amines and the consequent conjunction of a broad spectrum of lipophile and hydrophile characters to a quinoxaline ring system. 3-(3-benzyloxyquinoxalin-2-yl) propanhydrazide was produced in a two-step reaction of methyl 3-(3-oxo-3,4-dihydroquinoxalin-2-yl) propanoate with benzyl chloride followed by the hydrazinolysis of the corresponding ester. The antiproliferative activity of the compounds was tested in various cancer cell lines, including PC-3, Hela, HCT-116, and MCF-7; they showed a wide spectrum of activity for most of the tested compounds. Compound **6k** exhibited the highest activity, which was comparable to that of doxorubicin, with IC_50_ (µM) values of 12.17 ± 0.9, 9.46 ± 0.7, 10.88 ± 0.8, and 6.93 ± 0.4 µM compared to 8.87 ± 0.6, 5.57 ± 0.4, 5.23 ± 0.3, and 4.17 ± 0.2 µM for doxorubicin against Hela, HCT-116, and MCF-7, respectively. The in silico mechanistic study revealed the inhibition of HDAC-6 through the binding of the unique zinc finger ubiquitin-binding domain (HDAC6 Zf-UBD). The docking results showed a specific binding pattern that emphasized the crucial role of the quinoxaline ring and its substituents. The newly developed derivatives were evaluated for antitumor effects against four cancer cell lines PC-3, HeLa, HCT-116, and MCF-7. This research led to the identification of a quinoxaline-based scaffold exhibiting broad-spectrum antiproliferative activity and a distinct mechanism involving binding to HDAC6 Zf-UBD. The findings highlight its potential for further optimization and preclinical studies to support future anticancer drug development.

## 1. Introduction

**Figure 1:**
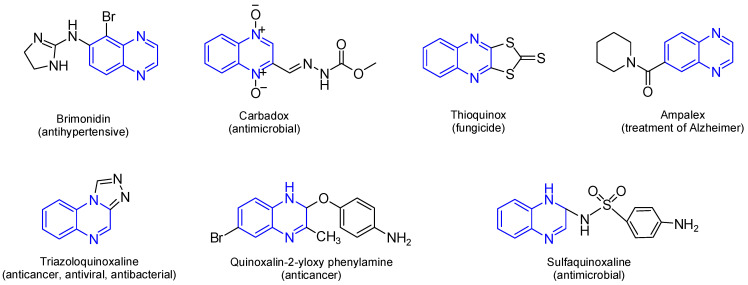
Bioactive and pharmaceutical of quinoxaline derivatives. Quinoxaline rings are highlighted in blue color.

Quinoxaline is a fused heterocyclic compound consisting of a benzene ring fused to a pyrazine ring. This privileged scaffold has attracted significant attention in medicinal chemistry due to its remarkable biological and pharmacological properties. Quinoxaline and its derivatives have been widely recognized for their diverse therapeutic applications, including anticancer [1,2,3], antitumor [4], antimicrobial, antiparasitic, antiviral, antioxidant [2,5], anti-inflammatory, anticonvulsant [6], and antidiabetic activities [5,7]. The structural rigidity and electron-rich nitrogen atoms of the quinoxaline ring contribute to its ability to interact with various biological targets, including enzymes, DNA, and receptor proteins, enhancing its therapeutic potential [8]. The pharmacological relevance of quinoxaline lies in its broad-spectrum activity and structural adaptability. Quinoxaline’s fused heterocyclic core can be readily modified at various positions to improve its pharmacological properties, enabling the development of potent and selective drug candidates [9]. The aromatic system of quinoxaline provides planarity, facilitating intercalation with nucleic acids or interaction with hydrophobic pockets in enzymes and receptors [10,11]. Moreover, the nitrogen atoms in the ring system play a critical role in forming hydrogen bonds and coordination complexes, which are essential for biological activity, further allowing it to intercalate into nucleic acids or engage with hydrophobic pockets in enzymes and receptors [7,12,13]. Additionally, several studies have shown a wide range of pharmacological activities for quinoxaline derivatives (Figure 1) [2,3,4,7,14,15,16,17]. There are many commercially available quinoxalines that play an essential role in the pharmaceutical and industrial market [18]. Several clinically approved drugs and therapeutic agents incorporate the quinoxaline moiety, for instance, Carbadox, a veterinary antimicrobial [14], and Echinomycin, a quinoxaline-based cyclic peptide antibiotic with antitumor activity [15,16]. Erdafitinib (Balversa), a quinoxaline containing pan-FGFR inhibitor, received full FDA approval for FGFR-altered advanced urothelial carcinoma [13].

Quinoxaline can be easily synthesized and structurally modified through various methods for pharmacokinetic improvement [10,19,20]. The quinoxaline scaffold is significant in anticancer drug development showing inhibition of topoisomerase enzymes [21,22,23,24], and, specific kinase inhibitors, especially those involved in cancer cell signaling path [25,26,27], antiproliferative effects against various cancer cell lines, and the induction of apoptosis [28,29,30,31,32]. Chemoselective reaction of amides with electrophiles is a well-known simple procedure for structure modification of biologically active compounds to produce either pure *N*, *O*-substituted products or a mixture of both [33,34,35,36,37]. We now report the synthesis of *N*-alkyl 3-(3-benzyloxyquinoxalin-2-yl) propanamides based on azide coupling of 3-(3-benzyloxyquinoxalin-2-yl) propanhydrazide with a variety of amines with a broad range of lipophilicity for biological evaluation.

## 2. Results and Discussion

### 2.1. Chemistry

Methyl 3-(3-oxo-3,4-dihydroquinoxalin-2-yl) propanoate (**1**) is considered as an excellent model for examining the chemoselctive reactions of amides with electrophiles and the structure modification of the quinoxaline ring for the biological evaluation. The chemoselective reactions of amides with electrophiles are governed by a number of factors. The structure of the parent amide and electrophiles in use, ruled by Hard-Soft Acid-Base Principle (HSAB Principle), other factors is related to solvent and bases [33,34,35,36,37]. This behavior directed the reaction of amides with electrophiles to produce one of the following three categories: *N*-substituted, *O*-substituted and a mixture of both *N-* and *O*-. Some researchers tend to block the harder nucleophile using very polar solvents and consequently directed the reaction to give selective softer alkylated product [38,39]. Another factor is the strong bases as NaH might affect the tautomerization equilibrium of the ambident amide and make the oxygen more available for the reaction with hard electrophiles [40,41]. After a number of attempts using a variety of solvents; quinoxaline **1** reacted benzyl chloride in the presence of potassium carbonate in DMF, acetonitrile and ethanol for 12 h. and gave a mixture of methyl 3-(4-benzyl-3-oxo-3,4-dihydroquinoxalin-2-yl) propanoate (**2**) and methyl 3-(3-benzyloxyquinoxalin-2-yl) propanoate (**3**) in a variety of yields, Table 1.

**Scheme 1:**
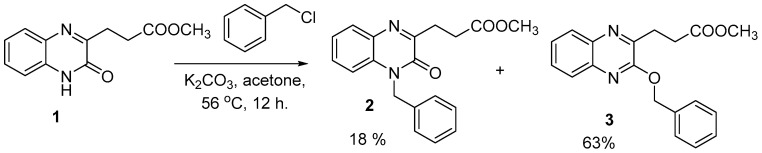
Reaction of quinoxaline (**1**) with benzyl chloride in acetone under reflux, yielding a mixture of compounds (**2**) (18%) and (**3**) (63%).

Targeting the selective formation of *O*-substituted methyl 3-(3-benzyloxyquinoxalin-2-yl) propanoate (**3**) or in best cases with higher yields. quinoxaline 1 reacted with benzyl chloride in the presence of dry potassium carbonate in acetone under reflux condition for 12 h. and gave a mixture of *N*- (**2**) and the desired *O*-benzylquinoxaline (**3**) in 18% and 63%, respectively, Scheme 1.

**Scheme 2:**
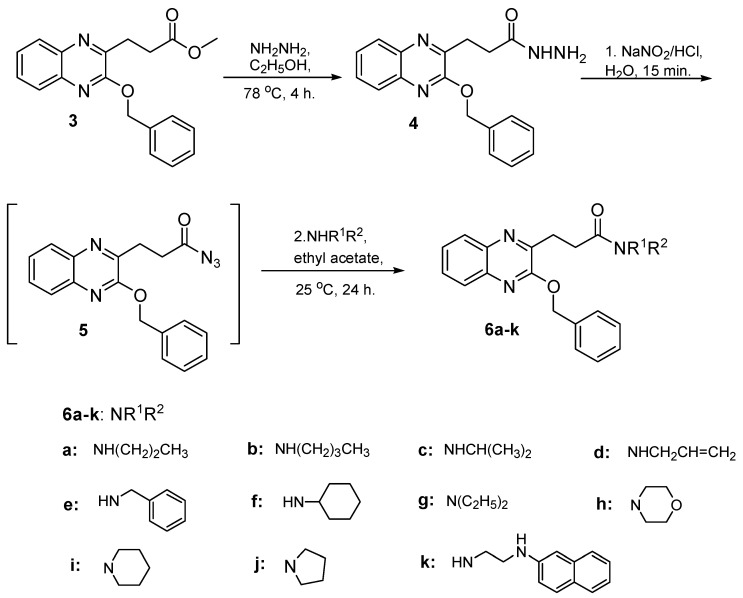
Preparation of *N*-alkyl 3-(3-benzyloxyquinoxalin-2-yl) propanamides **6a–k**.

*O*-Benzylquinoxaline **3** reacted with hydrazine hydrate in ethanol 95% for 4 h. gave 3-(3-benzyloxyquinoxalin-2-yl) propanhydrazide (**4**) in 87% yield, Scheme 2. Hydrazide **4** is a useful precursor for the structure modification of quinoxaline ring via azide coupling method to introduce a number of residues having lipophile and hydrophile characters useful for biological application. Thus, the reaction of hydrazide **4** with NaNO_2_ and HCl mixture at 0 °C presumably gave the azide **5** and was directly extracted with cold ethyl acetate, dried and used insitu without further purification. The in situ generated ethyl acetate azide **5** solution reacted with a variety of primary and secondary amines to afford *N*-alkyl 3-(3-benzyloxyquinoxalin-2-yl) propanamide **6a–k** in excellent yield, Scheme 2.

The structure assignment of methyl 3-(3-benzyloxyquinoxalin-2-yl) propanoate (**3**), 3-(3-benzyloxyquinoxalin-2-yl) propanhydrazide (**4**) and *N*-alkyl 3-(3-benzyloxyquinoxalin-2-yl) propanamides **6a–k** was based on ^1^H, ^13^C NMR as well as physicochemical analysis. Thus, the ^1^H NMR spectrum of *N*-allyl-3-(3-benzyloxyquinoxalin-2-yl)propanamide (**6d**) exhibits signals at δ 3.78, 4.96–5.09 and 5.67–5.74 ppm corresponding to NCH_2_, CH_2_=CH and CH_2_=CH, respectively of the allyl residue. The ^1^H NMR spectrum of (**6d**) also shows signals at δ 2.73, 3.25 and 5.45 ppm corresponding to CH_2_, CH_2_CO and OCH_2_, respectively. the ^13^C NMR spectrum of *N*-allyl-3-(3-benzyloxyquinoxalin-2-yl)propanamide (**6d**) showed signals at δ 45.0, 115.8 and 135.4 (CH) ppm due to NHCH_2_, CH_2_=CH and CH_2_=CH, respectively of the allyl residue, while gave signals at δ 27.9, 30.3, and 68.1 ppm due to CH_2_, CH_2_CO and OCH_2_, respectively, typically associated with *O*-substitution.

### 2.2. Cytotoxicity Assay

The compounds were tested in various cancer cell lines to explore their potential antiproliferative activity and their suitability for further testing and development. Four cancer types were initially selected including prostate (PC-3), ovarian (Hela), Colon (HCT-116), and breast (MCF-7). The results showed an interesting inhibition profile with a noticed relative activity against the tested cell lines. Compounds showed the highest activity against MCF-7, and Hela the relative lower activity against PC-3, and HCT-116. The IC_50_ ranged from 6.93 ± 0.4–85.20 ± 4.2 µM for MCF-7, 10.88 ± 0.8–81.65 ± 4.0 µM for HCT-116, 9.46 ± 0.7–91.38 ± 4.8 µM for Hela, and 12.17 ± 0.9–94.14 ± 4.9 µM for PC-3 (Table 2). Compound **6k** exhibited the highest activity against the four cancer cell lines with IC_50_ of 6.93 ± 0.4, 10.88 ± 0.8, 9.46 ± 0.7, and 12.17 ± 0.9 µM for MCF-7, HCT-116, Hela, and PC-3 respectively. In general, compounds that showed good activity (IC [42] < 50 µM) included compounds **2**, **4**, **6f**, **6g**, and **6i** Compounds with moderate to weak activity (IC_50_ 50–100 µM) included compounds **3**, **6a**, **6b**, **6c**, **6d**, **6e**, **6h**, and **6j**. In conclusion, the synthesized quinoxaline compounds showed good antiproliferative activity and wide spectrum of activity against the tested cell lines and deserve further investigations and development.

### 2.3. Docking Study

The synthesized quinoxaline derivatives exhibited a wide range of antiproliferative activity against various cancer types including prostate cancer (PC-3), ovarian cancer (Hela), colon cancer (HCT-116), and breast cancer (MCF-7). The initial structure activity elucidation revealed that the lead compounds **2** and **4** showed good activity compared to some of the substituted amide derivatives (**6k**, **6i**) while other derivatives exhibited lower activity (**6b**, **6e**). The compounds **6f**, **6h**, **6i**, and **6j** although bearing similar substituents, showed variable activity with **6i** being the most active followed by **6f**. Therefore, a more specific binding requirements should be investigated based on the stereochemical conformations and target interactions. Searching for similar structures of the lead compounds **3**, and **4** on PubChem revealed an experimentally validated target of structurally similar compounds. This target was identified as HDAC-6 bound to an inhibitor interestingly which binds the unique zinc finger ubiquitin-binding domain (HDAC6 Zf-UBD) and not the catalytic domain [42]. HDAC6 is localized in the cytoplasm has multifaceted functions in cell migration and reprogramming, and is associated with several diseases, including cancer and viral infections. Indeed, this was the first small molecule known to bind the HDAC6 Zf-UBD therefore, the development of other HDAC6 Zf-UBD inhibitors signifies an innovative strategy for elucidating the in vivo functions of HDAC-6 and the subsequent development of therapeutically active molecules to treat diseases such as cancer.

All compounds were docked in the HDAC6 Zf-UBD binding site using Glide in Maestro applying the extra precision (XP) module. The docking was initially validated by redocking the crystal ligand and computing the RMSD of the anticipated conformation relative to the crystal structure. The computed RMSD was determined to be 1.98, indicating good accuracy in the software’s prediction of docking poses of similar structures on the HDAC6 Zf-UBD binding site.

The XP docking scores were found to perfectly align with the experimental activity range of most of the tested compounds, categorizing the compounds into active, moderately active, weakly active, and inactive (Table 3). MM-GBSA enhances docking outcomes by integrating solvation and ligand flexibility, yielding more accurate binding free energy estimations. The results of the binding free energy calculations showed negative values for all compounds ranging from −44.99 to −29.60, which reflected favorable binding for the whole series. The calculations also stratified the compounds well relative to their experimental IC_50_. Compound **2** had the highest free energy of binding (−44.99 Kcal/mol) followed by compound **6k** (−42.76 Kcal/mol) and, compound **6g** (−37.05 Kcal/mol), while compound **3** showed the lowest free energy of binding (−29.60 Kcal/mol). Interestingly, the crystal ligand had a calculated binding free energy of −22.80 Kcal/mol which is even lower than the least active compounds (**3, 6b**) and almost half of the predicted activity of the most active compounds (**2, 6k**).

**Figure 2:**
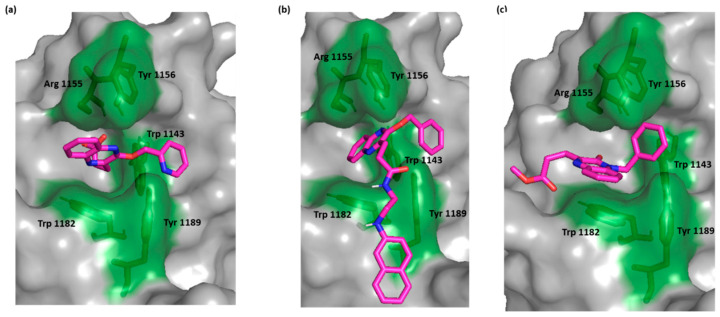
Surface representation of HDAC-6 (PDB IDs: 5WPB) binding site, showing key interacting residues in green sticks and bound to (**a**) crystal ligand, (**b**) compound **6k**, and (**c**) compound **4**.

For the most active compounds, **4** and **6k**, and similar to the crystal ligand, the compounds docked in the deep cavity pocket which normally accommodates the C-terminal residues of the C-terminal end of ubiquitin. For these compounds, the quinoxaline ring lay between Arg 1155 and Trp 1182 and established π-π stacking with Trp 1182. In the crystal ligand, a carboxylate group moved deeper into the cavity and formed a H bond with Arg 1155. This interaction was lacking in both compounds **6k** and **4** suggesting that the π-π stacking with Trp 1182 is more important than binding Arg 1155. The most active compound, as with the crystal ligand, was able to extend its benzyloxy group into the adjacent juxtaposed pocket and binds Trp 1143 (Figure 2).

**Figure 3:**
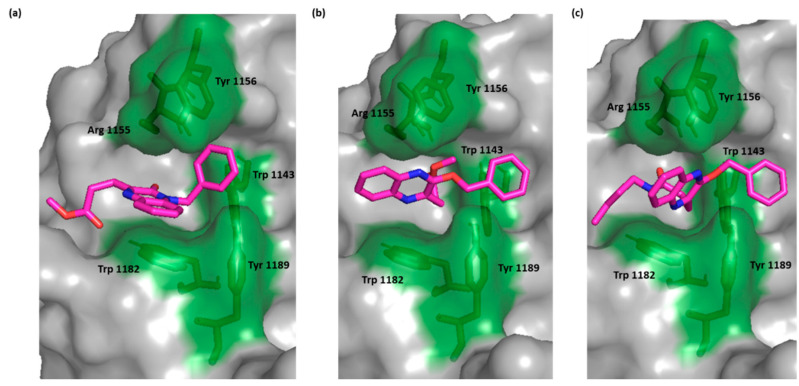
Surface representation of HDAC-6 (PDB IDs: 5WPB) binding site showing key interacting residues in green sticks and bound to (**a**) compound **2**, (**b**) compound **3**, (**c**) compound **6b** (green).

Compound **2**, a moderately active compound performed π-π stacking with Trp 1182 while, the low active compounds, **3** and **6b**, lacked this interaction while maintaining interaction with Trp 1143. This finding emphasized the importance of Trp 1182 as a crucial residue in binding the HDAC6 Zf-UBD binding site and through π interaction (Figure 3).

**Figure 4:**
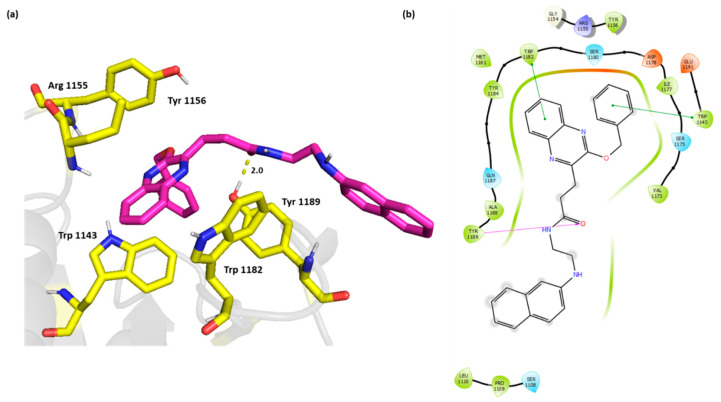
(**a**) Three-dimensional representation of the binding interactions between compound **6k** as sticks (carbon atoms in magenta), and HDAC-6 (PDB IDs: 5WPB) binding site shown as sticks (carbon atom in yellow). Potential electrostatic interactions are represented as yellow dotted lines and are measured in Angstrom. (**b**) Two-dimensional ligand–protein binding interactions between compound **6k** and HDAC-6 (PDB IDs: 5WPB) binding site. H bonds are represented as purple lines and π-π stacking as green lines.

The 2D and 3D overlay of compound **6k** is represented in Figure 4 On top of the π-π stacking with Trp 1182, and Trp 1143, the compound was able to bind Tyr 1189 through H bond. The naphthyl aminoethyl chain protruded outside the cavity and formed van der Waals interactions with Ser 1108, Pro 1109, and Leu 1110.

**Figure 5:**
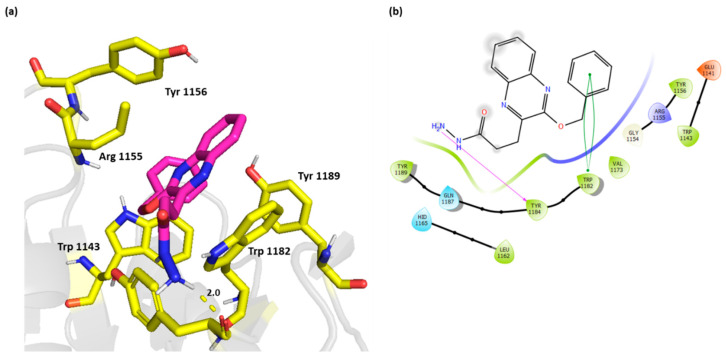
(**a**) Three-dimensional representation of the binding interactions between compound **4** as sticks (carbon atoms in magenta), and HDAC-6 (PDB IDs: 5WPB) binding site shown as sticks (carbon atom in yellow). Potential electrostatic interactions are represented as yellow dotted lines and are measured in Angstrom. (**b**) Two-dimensional ligand–protein binding interactions between compound **4** and HDAC-6 (PDB IDs: 5WPB) binding site. H bonds are represented as purple lines and π-π stacking as green lines.

Compound **4** on the other hand did not exhibit the π-π stacking with Trp 1143, however, it interacted with Tyr 1184 through H bond. This active compound showed a strong π-π interaction with Trp 1182 (Figure 5).

**Figure 6:**
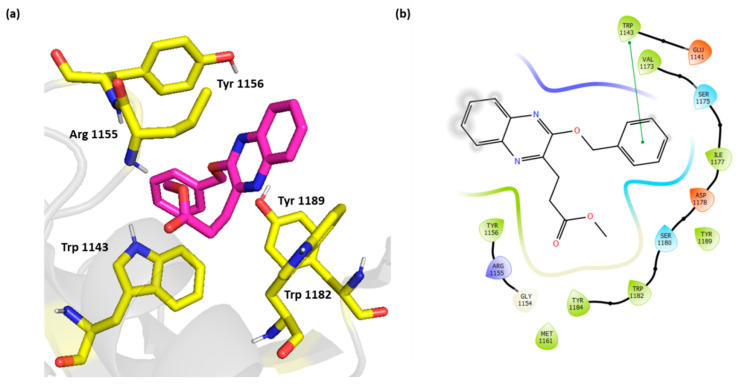
(**a**) Three-dimensional representation of the binding interactions between compound **3** as sticks (carbon atoms in magenta), and HDAC-6 (PDB IDs: 5WPB) binding site shown as sticks (carbon atom in yellow). Potential electrostatic interactions are represented as yellow dotted lines and are measured in Angstrom. (**b**) Two-dimensional ligand–protein binding interactions between compound **3** and HDAC-6 (PDB IDs: 5WPB) binding site. π-π stacking are represented as green lines.

The weakly active compound **3** showed only π-π stacking with its benzyloxy group and Trp 1143 with no additional electrostatic interactions (Figure 6).

**Figure 7:**
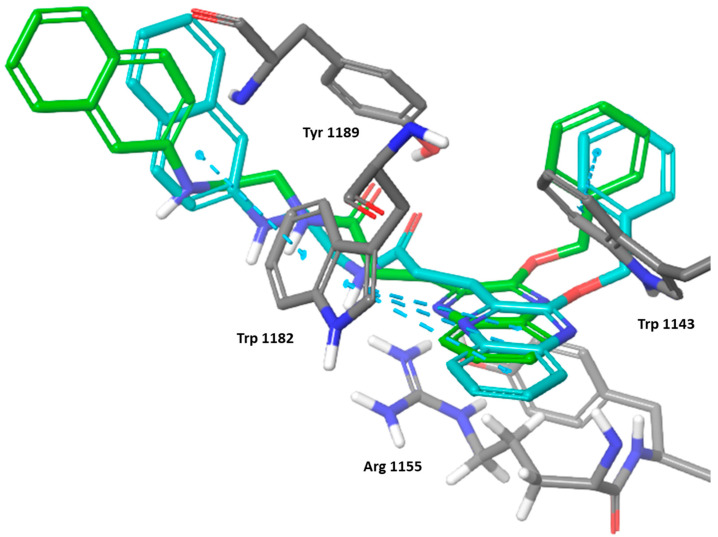
Three-dimensional representation of the binding interactions between compound **6k**; induced fit pose as sticks (carbon atoms in cyan), XP docking pose as sticks (carbon atoms in green), and HDAC-6 (PDB IDs: 5WPB) binding site shown as sticks (carbon atom in gray). π-π stacking are represented as dashed lines.

The induced fit docking for the most active compound **6k** showed an induced fit that strengthened the π-π stacking with Trp 1182 and Trp 1143 as well as potental hydrogen bonding with Arg 1155 (Figure 7). The IDF score was found to be −192.83 indicating a highly favorable binding interaction.

**Figure 8:**
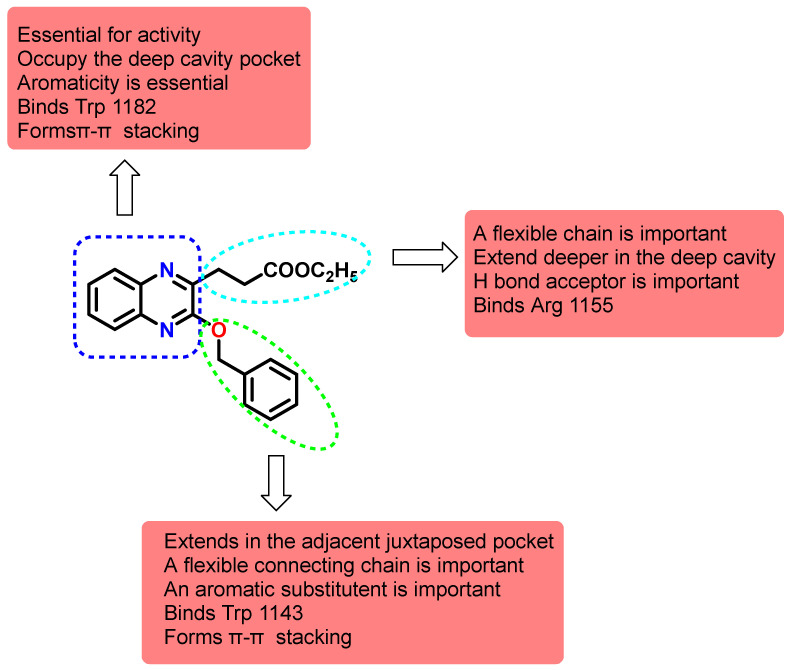
Structure activity relationship map of synthesized quinoxalines on HDAC6 Zf-UBD binding site.

A more conclusive structure activity relationship was depicted after the docking studies. The quinoxaline ring with its aromaticity is essential for activity to bind the key residues Trp 1182 through π-π stacking. Nevertheless, the importance of the heterocyclic nitrogen was not concluded which provided the potential for other heterocycles and benzoheterocycles. The substitution in the 2 and 3 positions are important to fulfil the other binding requirements that confer potency and selectivity for the compounds. Although the benzyloxy did not seem to be crucial since the oxygen is not involved in binding, importantly, the arylalkyl substituent should extend into the adjacent cavity and bind Trp 1143 through π-π stacking. The second substituent should bear a flexible chain to extend deeply into the cavity and H bond acceptor preferably a carboxylate that binds Arg 1155 through the H bonds or more strongly with a salt bridge (Figure 8).

In conclusion, the synthesized quinoxaline derivatives showed good in silico inhibitory activity to HDAC-6 through binding to the HDAC6 Zf-UBD binding site. The compounds represent initial leads that could be further optimized for binding and preclinical development. Future optimization should rely on design molecules that are tailored to occupy the ubiquitin cavity with the quinoxaline ring that binds to Trp 1182 and that bears two substituents one that would extend deeper into the cavity to bind Arg 1155 and another one with an aromatic system that would extend into the adjacent pocket and binds Trp 1143.

## 3. Materials and Methods

### 3.1. Chemistry

#### 3.1.1. General Procedures

All chemicals were obtained from Sigma-Aldrich., solvent were purified and dried in the usual way. Thin layer chromatography (TLC): silica gel 60 F_254_ plastic plates (E. Merck, layer thickness 0.2 mm) detected by UV absorption. Elemental analyses were performed on a Carlo Erba analyzer model 110 (Carlo Erba Instruments, Milan, Italy). Melting points were measured using a Stuart SMP10 digital melting point apparatus (Cole-Parmer Ltd., Stone, UK) and are reported without correction. ^1^H and ^13^C NMR spectra were recorded at 400 MHz, respectively (JEOL RESONANCE) in CDCl_3_ and DMSO solution with tetramethylsilane as an internal standard. The mass spectra were measured with a KRATOS analytical compact; on MALDI-MS the spectrometer was using 2,5-dihydroxybenzoic acid (DHB) as matrix. Methyl 3-(3-oxo-3,4-dihydroquinoxalin-2-yl) propanoate (**1**) was prepared according to reported literature [31,32].

#### 3.1.2. General Procedure for Alkylation of Methyl 3-(3-Oxo-3,4-dihydroquinoxalin-2-yl)propanoate (**1**)

Potassium carbonate (3.1 g, 22.5 mmol) and benzyl chloride (3.7 mL, 30 mmol) were added subsequently to a solution of methyl 3-(3-oxo-3,4-dihydroquinoxalin-2-yl) propanoate (**1**) (3.1 g, 22.5 mmol) in acetone (30 mL). For 12 h, the reaction mixture was heated to 56 °C. After the reaction mixture was filtered out, the filtrate was evaporated under reduced pressure, and the oily residue was column chromatographed using ethyl acetate: petroleum ether (3:1) as an eluent. This allowed for the eventual excellent yields of *N*- and *O*-allyl quinoxaline **2** and **3**, respectively.

##### Methyl 3-(4-Benzyl-3-oxo-3,4-dihydroquinoxalin-2-yl)propanoate (**2**)

White crystals (76%), M.p.: 132–133 °C. ^1^H NMR spectrum, (400 MHz, CDCl_3_), δ, ppm (*J*, Hz): 2.74 (t, *J* = 7.2 Hz, 2H, CH_2_), 3.36 (t, *J* = 7.2 Hz, 2H, CH_2_CO), 3.73 (s, 3H, OCH_3_), 5.50 (s, 2H, NCH_2_), 7.23–7.33 (m, 7H, Ar-H), 7.38–7.42 (m, 1H, Ar-H), 7.79 (d, *J* = 8.0 Hz, 1H, Ar-H). ^13^C NMR (400 MHz, CDCl_3_), δ, ppm: 27.9 (CH_2_), 30.3 (CH_2_CO), 45.9 (NHCH_2_), 53.8 (OCH_3_), 126.5 (C-Ar), 126.8, 127.0, 128.1, 128.5, 128.6, 128.7, 129.1, 136.6, 138.6, 139.8 (C-Ar), 148.8 (C=N), 155.5 (C=O), 173.4 (C=O). MS (MALDI, positive mode, matrix DHB): *m*/*z* = 345.3 (M + Na)^+^. Anal. Calcd. For C_19_H_18_N_2_O_3_ (322.37): C, 70.79; H, 5.63; N, 8.69. Found: C, 71.05; H, 5.41; N, 8.98.

##### Methyl 3-(3-Benzyloxyquinoxalin-2-yl)propanoate (**3**)

White crystals (63%), M.p.: 85–86 °C. ^1^H NMR spectrum, (400 MHz, CDCl_3_), δ, ppm (*J*, Hz): 2.98 (t, *J* = 7.2 Hz, 2 H, CH_2_), 3.38 (t, *J* = 7.2 Hz, 2 H, CH_2_CO), 3.75 (s, 3 H, OCH_3_), 5.61 (s, 2 H, OCH_2_), 7.36–7.47 (m, 3 H, Ar-H), 7.53–7.59 (m, 3 H, Ar-H), 7.65 (t, *J* = 8.0 Hz, 1 H, Ar-H), 7.83 (d, *J* = 8.0 Hz, 1 H, Ar-H), 7.98 (d, *J* = 8.0 Hz, 1 H, Ar-H). ^13^C NMR (400 MHz, CDCl_3_), δ, ppm: 27.1 (CH_2_), 30.7 (CH_2_CO), 51.3 (OCH_3_), 68.9 (OCH_2_), 126.5, 126.8, 126.9, 128.1, 128.5, 128.6, 128.7, 129.1, 136.6, 138.6, 139.8 (C-Ar), 148.2 (C=N), 155.6 (C=N), 173.7 (C=O). MS (MALDI, positive mode, matrix DHB): *m*/*z* = 345.3 (M + Na)^+^. Anal. Calcd. For C_19_H_18_N_2_O_3_ (322.1): C, 70.79; H, 5.63; N, 8.69. Found: C, 70.53; H, 5.83; N, 8.61.

#### 3.1.3. Preparation of 3-(3-Benzyloxyquinoxalin-2-yl)propanhydrazide (**4**)

After adding hydrazine hydrate (60%) (0.2 mL, 2.0 mmol) to a solution of methyl 3-(3-benzyloxyquinoxalin-2-yl) propanoate (**3**) (1.0 mmol) in ethyl alcohol 95% (15 mL), the reaction mixture was refluxed for 4 h, refrigerated for 12 h, and the resulting crystals were filtered and crystallized from ethanol 95% to yield **4**.

White crystals (87%), M.p.: 171–173 °C. ^1^H NMR spectrum, (400 MHz, DMSO), δ, ppm (*J*, Hz): 2.99 (t, *J* = 7.2 Hz, 2 H, CH_2_), 3.37 (t, *J* = 7.2 Hz, 2 H, CH_2_CO), 4.22 (bs, 2 H, NH_2_), 5.61 (s, 2 H, OCH_2_), 7.39–7.49 (m, 3 H, Ar-H), 7.55–7.59 (m, 3 H, Ar-H), 7.65 (t, *J* = 8.0 Hz, 1 H, Ar-H), 7.89 (d, *J* = 8.0 Hz, 1 H, Ar-H), 8.00 (d, *J* = 8.0 Hz, 1 H, Ar-H), 9.18 (bs, 1 H, NH). ^13^C NMR (400 MHz, DMSO), δ, ppm: 27.9 (CH_2_), 30.8 (CH_2_CO), 68.1 (OCH_2_), 126.5, 126.8, 126.9, 128.1, 128.6, 128.8, 128.9, 129.1, 136.6, 138.8, 139.7 (C-Ar), 148.7 (C=N), 155.6 (C=N), 170.5 (C=O). MS (MALDI, positive mode, matrix DHB): *m*/*z* = 345.3 (M + Na)^+^. Anal. Calcd. For C_18_H_18_N_4_O_2_ (322.37): C, 67.07; H, 5.63; N, 17.38. Found: C, 66.85; H, 5.43; N, 17.71.

#### 3.1.4. Preparation of *N*-Alkyl 3-(3-Benzyloxyquinoxalin-2-yl)propanamide **6a–k**

AcOH (6 mL), 1 N HCl (3 mL), and water (3 mL) were added to 3-(3-benzyloxyquinoxalin-2-yl) propanhydrazide (**4**) (1.0 g, 10.0 mmol) to create a slurry, which was then cooled to −5 °C for 15 min. A cold solution of NaNO_2_ (1.0 g, 15 mmol) in water (2.0 mL) was added to the slurry portion-wise for 20 min, resulting in a yellow syrup that was extracted multiple times using cold ethyl acetate (30 mL). The combined extracts were then rinsed with cold 3% NaHCO_3_, H_2_O, and finally dried (Na_2_SO_4_) to produce the in-situ generated ethyl acetate solution of azide **5**. Propyl amine, butyl amine, isopropyl amine, allyl amine, benzyl amine, cyclohexyl amine, diethyl amine, morpholine, piperidine, pyrrolidine and *N*^1^-(naphthalen-2-yl)ethane-1,2-diamine (10.0 mmol) were added to cold ethyl acetate solution of azide **5** and the reaction mixture was kept at −5 °C for 24 h. The reaction mixture was then washed with 1 N HCl, followed by a 3% solution of NaHCO_3_, H_2_O, and finally dried over (Na_2_SO_4_). The reaction mixture was evaporated under reduced pressure until it was completely dry, and the residue was crystallized with petroleum ether/ethyl acetate to yield the desired products **6a**–**k** in high yields.

##### 3-(3-Benzyloxyquinoxalin-2-yl)-*N*-propyl-propanamide (**6a**)

From *n*-propyl amine with 3-(3-benzyloxyquinoxalin-2-yl)propanoyl azide (**5**). White crystals (76%), M.p.: 101–103 °C. ^1^H NMR spectrum, (400 MHz, CDCl_3_), δ, ppm (*J*, Hz): 0.87–0.93 (m, 3 H, CH_3_), 1.45–1.54 (m, 2 H, CH_2_), 2.81 (t, *J* = 7.2 Hz, 2 H, CH_2_), 3.21 (q, *J* = 7.2 Hz, 2 H, NHCH_2_), 3.35 (t, *J* = 7.2 Hz, 2 H, CH_2_CO), 5.57 (s, 2 H, OCH_2_), 6.30 (bs, 1 H, NH), 7.33–7.42 (m, 3 H, Ar-H), 7.50–7.59 (m, 3 H, Ar-H), 7.63 (t, *J* = 8.0 Hz, 1 H, Ar-H), 7.85 (d, *J* = 8.0 Hz, 1 H, Ar-H), 7.93 (d, *J* = 8.0 Hz, 1 H, Ar-H). ^13^C NMR (400 MHz, CDCl_3_), δ, ppm: 14.2 (CH_3_), 23.1 (CH_2_), 27.9 (CH_2_), 27.9 (CH_2_), 30.7 (CH_2_CO), 40.2 (NHCH_2_), 68.1 (OCH_2_), 126.4, 126.8, 126.9, 128.1, 128.4, 128.6, 128.7, 129.1, 136.6, 138.6, 139.8 (C-Ar), 148.3 (C=N), 155.6 (C=N), 173.7 (C=O). MS (MALDI, positive mode, matrix DHB): *m*/*z* = 372.4 (M + Na)^+^. Anal. Calcd. For C_21_H_23_N_3_O_2_ (349.43): C, 72.18; H, 6.63; N, 12.03. Found: C, 72.11; H, 6.84; N, 11.86.

##### 3-(3-Benzyloxyquinoxalin-2-yl)-*N*-butyl-propanamide (**6b**)

From butyl amine with 3-(3-(benzyloxyquinoxalin-2-yl)propanoyl azide (**5**). White crystals (91%), M.p.: 85–86 °C. ^1^H NMR spectrum, (400 MHz, CDCl_3_), δ, ppm (*J*, Hz): 0.89 (t, *J* = 7.2 Hz, 3 H, CH_3_), 1.09–1.13 (m, 2 H, CH_2_), 1.31–1.36 (m, 2 H, CH_2_), 2.65 (t, *J* = 7.2 Hz, 2H, CH_2_), 3.15–3.22 (m, 4 H, NHCH_2_, CH_2_CO), 5.40 (s, 2 H, OCH_2_), 6.30 (bs, 1 H, NH), 7.14–7.24 (m, 3 H, Ar-H), 7.34–7.37 (m, 3 H, Ar-H), 7.49 (t, *J* = 8.0 Hz, 1H, Ar-H), 7.67 (d, *J* = 8.0 Hz, 1H, Ar-H), 7.74 (d, *J* = 8.0 Hz, 1H, Ar-H). ^13^C NMR (400 MHz, CDCl_3_), δ, ppm: 14.2 (CH_3_), 19.9 (CH_2_), 27.9 (CH_2_), 30.3 (CH_2_CO), 34.9 (CH_2_), 43.4 (NHCH_2_), 68.1 (OCH_2_), 126.5, 126.8, 126.9, 128.1, 128.5, 128.6, 128.7, 129.1, 136.6, 138.6, 139.7 (C-Ar), 148.8 (C=N), 155.6 (C=N), 171.3 (C=O). MS (MALDI, positive mode, matrix DHB): *m*/*z* = 386.4 (M + Na)^+^. Anal. Calcd. For C_22_H_25_N_3_O_2_ (363.46): C, 72.70; H, 6.93; N, 11.56. Found: C, 73.03; H, 7.21; N, 11.76.

##### 3-(3-Benzyloxyquinoxalin-2-yl)-*N*-isopropylpropanamide (**6c**)

From isopropyl amine with 3-(3-benzyloxyquinoxalin-2-yl)propanoyl azide (**5**). White crystals (67%), M.p.: 170–171 °C. ^1^H NMR spectrum, (400 MHz, CDCl_3_), δ, ppm (*J*, Hz): 1.02 (d, *J* = 6.0 Hz, 6 H, 2CH_3_), 2.68 (t, *J* = 7.2 Hz, 2 H, CH_2_), 3.27 (t, *J* = 7.2 Hz, 2 H, CH_2_CO), 3.92–4.00 (m, 1 H, NHCH), 5.48 (s, 2 H, OCH_2_), 6.00 (bs, 1 H, NH), 7.24–7.35 (m, 3 H, Ar-H), 7.44–7.48 (m, 3 H, Ar-H), 7.52–7.56 (m, 1 H, Ar-H), 7.78 (d, *J* = 8.0 Hz, 1 H, Ar-H), 7.86 (d, *J* = 8.0 Hz, 1 H, Ar-H). ^13^C NMR (400 MHz, CDCl_3_), δ, ppm: 23.1 (CH_3_), 27.9 (CH_2_), 30.3 (CH_2_CO), 48.7 (NHCH), 68.1 (OCH_2_), 126.5, 126.8, 126.9, 128.1, 128.5, 128.6, 128.7, 129.1, 136.7, 138.9, 139.7 (C-Ar), 148.8 (C=N), 155.6 (C=N), 172.5 (C=O). MS (MALDI, positive mode, matrix DHB): *m*/*z* = 372.4 (M + Na)^+^. Anal. Calcd. For C_21_H_23_N_3_O_2_ (349.43): C, 72.18; H, 6.63; N, 12.03. Found: C, 71.86; H, 6.85; N, 11.74.

##### *N*-Allyl-3-(3-(Benzyloxyquinoxalin-2-yl)propanamide (**6d**)

From allyl amine with 3-(3-benzyloxyquinoxalin-2-yl)propanoyl azide (**5**). White crystals (71%), M.p.: 67–68 °C. ^1^H NMR spectrum, (400 MHz, CDCl_3_), δ, ppm (*J*, Hz): 2.73 (t, *J* = 7.2 Hz, 2 H, CH_2_), 3.25 (t, *J* = 7.2 Hz, 2 H, CH_2_CO), 3.78 (t, *J* = 7.2 Hz, 2 H, NHCH_2_), 4.96–5.09 (m, 2 H, CH_2_=CH), 5.45 (s, 2 H, OCH_2_), 5.67–5.74 (m, 1 H, CH_2_=CH), 6.27 (bs, 1 H, NH), 7.23–7.32 (m, 3 H, Ar-H), 7.40–7.45 (m, 3 H, Ar-H), 7.53 (t, *J* = 8.0 Hz, 1 H, Ar-H), 7.76 (d, *J* = 8.0 Hz, 1 H, Ar-H), 7.89 (d, *J* = 8.0 Hz, 1 H, Ar-H). ^13^C NMR (400 MHz, CDCl_3_), δ, ppm: 27.9 (CH_2_), 30.3 (CH_2_CO), 45.0 (NHCH_2_), 68.1 (OCH_2_), 115.8 (CH_2_), 126.5, 126.8, 126.9, 128.1, 128.5, 128.6, 128.7, 129.1 (C-Ar), 135.4 (CH), 136.6, 138.7, 139.8 (C-Ar), 148.8 (C=N), 155.6 (C=N), 172.3 (C=O). MS (MALDI, positive mode, matrix DHB): *m*/*z* = 370.4 (M + Na)^+^. Anal. Calcd. For C_21_H_21_N_3_O_2_ (347.42): C, 72.60; H, 6.09; N, 12.10. Found: C, 72.87; H, 5.85; N, 11.92.

##### *N*-Benzyl-3-(3-Benzyloxyquinoxalin-2-yl)propanamide (**6e**)

From benzyl amine with 3-(3-benzyloxyquinoxalin-2-yl)propanoyl azide (**5**). White crystals (82%), M.p.: 122–123 °C. ^1^H NMR spectrum, (400 MHz, CDCl_3_), δ, ppm (*J*, Hz): 2.76 (t, *J* = 7.2 Hz, 2 H, CH_2_), 3.28 (t, *J* = 7.2 Hz, 2 H, CH_2_CO), 4.34 (d, *J* = 7.2 Hz, 2 H, NHCH_2_), 5.48 (s, 2 H, OCH_2_), 6.39 (bs, 1 H, NH), 7.21–7.35 (m, 4 H, Ar-H), 7.42–7.47 (m, 4 H, Ar-H), 7.52 (t, *J* = 8.0 Hz, 2 H, Ar-H), 7.74 (d, *J* = 8.0 Hz, 2 H, Ar-H), 7.86 (d, *J* = 8.0 Hz, 2 H, Ar-H). ^13^C NMR (400 MHz, CDCl_3_), δ, ppm: 27.9 (CH_2_), 30.3 (CH_2_CO), 45.9 (NHCH_2_), 68.1 (OCH_2_), 126.5, 126.8, 126.9, 127.4, 128.1, 128.5, 128.6, 128.7, 129.1, 129.4, 136.6, 137.1, 138.6, 139.8 (C-Ar), 148.9 (C=N), 155.6 (C=N), 172.9 (C=O). MS (MALDI, positive mode, matrix DHB): *m*/*z* = 420.4 (M + Na)^+^. Anal. Calcd. For C_25_H_23_N_3_O_2_ (397.48): C, 75.55; H, 5.83; N, 10.57. Found: C, 75.32; H, 6.03; N, 10.74.

##### 3-(3-Benzyloxyquinoxalin-2-yl)-*N*-cyclohexylpropanamide (**6f**)

From cyclohexyl amine with 3-(3-benzyloxyquinoxalin-2-yl)propanoyl azide (**5**). White crystals (66%), M.p.: 81–83 °C. ^1^H NMR spectrum, (400 MHz, CDCl_3_), δ, ppm (*J*, Hz): 1.18–1.30 (m, 2 H, CH_2_), 1.59–1.64 (m, 4 H, 2CH_2_), 1.79–1.85 (m, 4 H, 2CH_2_), 2.70 (t, *J* = 7.2 Hz, 2 H, CH_2_), 3.27 (t, *J* = 7.2 Hz, 2 H, CH_2_CO), 3.56–3.67 (m, 1 H, NHCH), 5.49 (s, 2 H, OCH_2_), 6.22 (bs, 1 H, NH), 7.24–7.35 (m, 3 H, Ar-H), 7.39–7.49 (m, 3 H, Ar-H), 7.53 (t, *J* = 8.0 Hz, 1 H, Ar-H), 7.76 (d, *J* = 8.0 Hz, 1 H, Ar-H), 7.84 (d, *J* = 8.0 Hz, 1 H, Ar-H). ^13^C NMR (400 MHz, CDCl_3_), δ, ppm: 23.1 (CH_2_), 25.3 (CH_2_), 27.8 (CH_2_), 30.3 (CH_2_CO), 32.8 (CH_2_), 53.8 (NHCH), 68.1 (OCH_2_), 126.5, 126.8, 126.9, 128.1, 128.5, 128.6, 128.7, 129.1 (C-Ar), 136.6, 138.6, 139.8 (C-Ar), 148.9 (C=N), 155.7 (C=N), 172.8 (C=O). MS (MALDI, positive mode, matrix DHB): *m*/*z* = 412.5 (M + Na)^+^. Anal. Calcd. For C_24_H_27_N_3_O_2_ (389.2): C, 74.01; H, 6.99; N, 10.79. Found: C, 73.79; H, 7.21; N, 10.98.

##### 3-(3-Benzyloxyquinoxalin-2-yl)-*N*,*N*-diethylpropanamide (**6g**)

From diethyl amine with 3-(3-benzyloxyquinoxalin-2-yl)propanoyl azide (**5**). White crystals (77%), M.p.: 96–97 °C. ^1^H NMR spectrum, (400 MHz, CDCl_3_), δ, ppm (*J*, Hz): 1.13 (t, *J* = 7.2 Hz, 3 H, CH_3_), 1.25 (t, *J* = 7.2 Hz, 3 H, CH_3_), 2.92 (t, *J* = 7.2 Hz, 2 H, CH_2_), 3.39–3.43 (m, 6 H, CH_2_CO, 2NCH_2_), 5.59 (s, 2 H, OCH_2_), 7.23–7.38 (m, 3 H, Ar-H), 7.49–7.56 (m, 4 H, Ar-H), 7.83 (d, *J* = 8.0 Hz, 1 H, Ar-H), 7.97 (d, *J* = 8.0 Hz, 1 H, Ar-H). ^13^C NMR (400 MHz, CDCl_3_), δ, ppm: 14.2 (2CH_3_), 27.9 (CH_2_), 30.3 (CH_2_CO), 45.0 (2NCH_2_), 68.1 (OCH_2_), 126.5, 126.8, 126.9, 128.1, 128.5, 128.6, 128.7, 129.1, 136.6, 138.7, 139.7 (C-Ar), 148.8 (C=N), 155.7 (C=N), 172.6 (C=O). MS (MALDI, positive mode, matrix DHB): *m*/*z* = 386.4 (M + Na)^+^. Anal. Calcd. For C_22_H_25_N_3_O_2_ (363.46): C, 72.70; H, 6.93; N, 11.56. Found: C, 72.43; H,7.23; N, 11.69.

##### 3-(3-Benzyloxyquinoxalin-2-yl)-1-morpholin-4-yl-propan-1-one (**6h**)

From morpholine with 3-(3-benzyloxyquinoxalin-2-yl)propanoyl azide (**5**). White crystals (64%), M.p.: 86–87 °C. ^1^H NMR spectrum, (400 MHz, CDCl_3_), δ, ppm (*J*, Hz): 2.89 (t, *J* = 7.2 Hz, 2 H, CH_2_), 3.36 (t, *J* = 7.2 Hz, 2 H, CH_2_CO), 3.47–3.72 (m, 8 H, 2OCH_2_, 2NCH_2_), 5.58 (s, 2 H, OCH_2_), 7.24–7.41 (m, 3 H, Ar-H), 7.50–7.58 (m, 3 H, Ar-H), 7.61 (t, *J* = 8.0 Hz, 1H, Ar-H), 7.83 (d, *J* = 8.0 Hz, 1H, Ar-H), 7.97 (d, *J* = 8.0 Hz, 1H, Ar-H). ^13^C NMR (400 MHz, CDCl_3_), δ, ppm: 27.9 (CH_2_), 30.8 (CH_2_CO), 46.3 (2NCH_2_), 65.1 (2OCH_2_), 68.1 (OCH_2_), 126.5, 126.6, 126.9, 128.1, 128.5, 128.6, 128.9, 129.1, 136.6, 138.7, 139.8 (C-Ar), 148.8 (C=N), 155.5 (C=N), 172.6 (C=O). MS (MALDI, positive mode, matrix DHB): *m*/*z* = 400.4 (M + Na)^+^. Anal. Calcd. For C_22_H_23_N_3_O_3_ (377.44): C, 70.01; H, 6.14; N, 11.13. Found: C, 69.81; H, 6.43; N, 10.88.

##### 3-(3-Benzyloxyquinoxalin-2-yl)-1-piperidin-1-yl-propan-1-one (**6i**)

From piperidine with 3-(3-benzyloxyquinoxalin-2-yl)propanoyl azide (**5**). White crystals (56%), M.p.: 74–75 °C. ^1^H NMR spectrum, (400 MHz, CDCl_3_), δ, ppm (*J*, Hz): 1.23–1.30 (m, 2 H, CH_2_), 1.52–1.67 (m, 4 H, 2CH_2_), 2.91 (t, *J* = 7.2 Hz, 2 H, CH_2_), 3.36 (t, *J* = 7.2 Hz, 2 H, CH_2_CO), 3.47–3.58 (m, 4 H, 2NCH_2_), 5.58 (s, 2 H, OCH_2_), 7.21–7.41 (m, 3 H, Ar-H), 7.50–7.56 (m, 3 H, Ar-H), 7.61 (t, *J* = 8.0 Hz, 1 H, Ar-H), 7.84 (d, *J* = 8.0 Hz, 1 H, Ar-H), 7.96 (d, *J* = 8.0 Hz, 1 H, Ar-H). ^13^C NMR (400 MHz, CDCl_3_), δ, ppm: 21.4 (CH_2_), 25.4 (CH_2_), 27.9 (CH_2_), 30.3 (CH_2_CO), 46.3 (2NCH_2_), 68.1 (OCH_2_), 126.5, 126.8, 126.9, 128.1, 128.5, 128.6, 128.7, 129.1, 136.6, 138.7, 139.8 (C-Ar), 148.8 (C=N), 155.9 (C=N), 172.4 (C=O). MS (MALDI, positive mode, matrix DHB): *m*/*z* = 398.4 (M + Na)^+^. Anal. Calcd. For C_23_H_25_N_3_O_2_ (375.47): C, 73.57; H, 6.71; N, 11.19. Found: C, 73.22; H, 6.43; N, 10.78.

##### 3-(3-Benzyloxyquinoxalin-2-yl)-1-(pyrrolidin-1-yl)propan-1-one (**6j**)

From pyrrolidine with 3-(3-benzyloxyquinoxalin-2-yl)propanoyl azide (**5**). White crystals (74%), M.p.: 67–68 °C. ^1^H NMR spectrum, (400 MHz, CDCl_3_), δ, ppm (*J*, Hz): 1.74 (t, *J* = 7.2 Hz, 2 H, CH_2_), 1.84 (t, *J* = 7.2 Hz, 2 H, CH_2_), 2.75 (t, *J* = 7.2 Hz, 2 H, CH_2_), 3.28 (t, *J* = 7.2 Hz, 2 H, NCH_2_), 3.36 (t, *J* = 7.2 Hz, 2 H, CH_2_CO), 3.42 (t, *J* = 7.2 Hz, 2 H, NCH_2_), 5.45 (s, 2 H, OCH_2_), 7.20–7.39 (m, 3 H, Ar-H), 7.40–7.51 (m, 4 H, Ar-H), 7.75 (d, *J* = 8.0 Hz, 1 H, Ar-H), 7.87 (d, *J* = 8.0 Hz, 1 H, Ar-H). ^13^C NMR (400 MHz, CDCl_3_), δ, ppm: 26.2 (2CH_2_), 27.9 (CH_2_), 30.3 (CH_2_CO), 48.7 (2NCH_2_), 68.1 (OCH_2_), 126.5, 126.8, 126.9, 128.1, 128.5, 128.6, 128.7, 129.1, 136.7, 138.7, 139.8 (C-Ar), 148.8 (C=N), 155.9 (C=N), 171.3 (C=O). MS (MALDI, positive mode, matrix DHB): *m*/*z* = 384.4 (M + Na)^+^. Anal. Calcd. For C_22_H_23_N_3_O_2_ (361.44): C, 73.11; H, 6.41; N, 11.63. Found: C, 73.53; H, 6.21; N, 11.82.

##### 3-(3-Benzyloxyquinoxalin-2-yl)-*N*-(2-(naphthalen-2-ylamino)ethyl)propanamide (**6k**)

From *N*^1^-(naphthalen-2-yl)ethane-1,2-diamine with 3-(3-benzyloxyquinoxalin-2-yl)-propanoyl azide (**5**). White crystals (71%), M.p.: 148–149 °C. ^1^H NMR spectrum, (400 MHz, CDCl_3_), δ, ppm (*J*, Hz): 2.76–3.79 (m, 2 H, CH_2_), 3.25–3.29 (m, 2 H, CH_2_CO), 3.57–3.65 (m, 4 H, 2 NHCH_2_), 5.49 (s, 2 H, OCH_2_), 6.32 (bs, 1 H, NH), 6.62 (bs, 1 H, NH), 7.08–7.32 (m, 9 H, Ar-H), 7.41–7.69 (m, 7 H, Ar-H). ^13^C NMR (400 MHz, CDCl_3_), δ, ppm: 27.9 (CH_2_), 30.3 (CH_2_CO), 40.2 (NHCH_2_), 43.4 (NHCH_2_), 68.1 (OCH_2_), 122.5, 123.7, 125.7, 126.5, 126.8, 126.9, 127.4, 127.7, 128.1, 128.5, 128.6, 128.7, 129.1, 129.4, 130.2, 131.6, 136.7, 138.6, 139.8, 142.9 (C-Ar), 148.8 (C=N), 155.5 (C=N), 170.8 (C=O). MS (MALDI, positive mode, matrix DHB): *m*/*z* = 499.5 (M + Na)^+^. Anal. Calcd. For C_30_H_28_N_4_O_2_ (476.58): C, 75.61; H, 5.92; N, 11.76. Found: C, 75.32; H, 6.22; N, 11.54.

### 3.2. Biological Studies

#### 3.2.1. Cell Line

Human prostate cancer (PC3), Epitheliod Carcinoma Cervix cancer (Hela), Mammary gland breast cancer (MCF-7) and Colorectal carcinoma Colon cancer (HCT-116). The cell line was obtained from ATCC via Holding company for biological products and vaccines (VACSERA).

#### 3.2.2. Reagents and Cell Viability Assay

The experimental materials included RPMI-1640 culture medium, MTT reagent, and dimethyl sulfoxide (DMSO), all sourced from Sigma-Aldrich (St. Louis, MO, USA), along with fetal bovine serum (FBS) obtained from GIBCO (UK Paisley, UK). Doxorubicin was employed as the standard reference drug for evaluating anticancer activity. To assess the cytotoxic effects of the synthesized compounds, an MTT assay was performed on the previously described cell lines. This colorimetric technique depends on the enzymatic reduction of the yellow tetrazolium bromide (MTT) to a purple formazan product by mitochondrial dehydrogenase enzymes in viable cells. Cells were cultured in RPMI-1640 medium supplemented with 10% FBS, 100 U/mL of penicillin, and 100 µg/mL of streptomycin. They were incubated at 37 °C in a humidified environment containing 5% carbon dioxide. For the assay, cells were seeded into 96-well plates at a density of 1 × 10^4^ cells per well and allowed to adhere for 48 h. Subsequently, they were treated with various concentrations of the test compounds and incubated for 24 h. Following treatment, 20 µL of MTT solution (5 mg/mL) was added to each well and the plates were incubated for an additional 4 h to allow for formazan crystal formation. After incubation, 100 µL of DMSO was added to each well to dissolve the formazan. The absorbance was measured at 570 nm using a microplate reader (EXL 800, BioTek Instruments, Winooski, VT, USA). Cell viability was the percentage of relative cell viability was determined by the equation: (A_570_ of treated cells/A_570_ of untreated control) × 100 [43,44].

### 3.3. Molecular Modeling and Docking

#### 3.3.1. Software

The molecular modelling software Maestro by Schrödinger (Version 2024-4 software release) was used for computational studies [45].

#### 3.3.2. Crystal Structures

The X-ray coordinates of HDAC-6 in complex with inhibitor in the zinc finger domain and Ubiquitin (PDB IDs: 5WPB) was obtained from the Research Collaboratory for Structural Bioinformatics (RCSB) Protein Data Bank (PDB) [46,47].

#### 3.3.3. Protein Preparation

The PDB structures were optimized for docking via the Protein Preparation Workflow. The preparation and minimizing process occurred at a pH of 7.4, with adjustments made to the ionization states. Polar hydrogens were included, while non-essential water molecules were eliminated from the structures. The structures of the targets were ultimately minimized using the OPLS3 force field, with a preset rmsd value of 0.30 Å for non-hydrogen atoms [48].

#### 3.3.4. Receptor Grid Generation

A grid box of 20 Å^3^ was generated at the centre of the bound ligand without any constraints and with a 1.00 van der Waals radius along with a cutoff of 0.25 for partial charges using default parameters.

#### 3.3.5. Ligand Preparation

The ligands were prepared using LigPrep, by generating the most likely ionization states at a pH of 7 ± 1 and then structures’ optimization with the OPLS3 force field.

#### 3.3.6. Validation of the Molecular Docking

The validation of the molecular docking was performed by calculating the accuracy of the predicted conformations’ match with the experimental conformations [49,50,51]. The crystallographic ligand was docked into the protein target using the same docking criteria used for the prepared ligands. The docked position with the lowest binding energy was then aligned with the conformation of the crystallographic structure using Maestro’s structure superimposition tool and the root mean square deviation (RMSD) of the alignment was then calculated.

#### 3.3.7. Molecular Docking

The extra precision mode (XP) on Glide was employed for all docking operations, utilizing a van der Waals (vdw) radius scaling factor of 0.80, a partial charge cut-off of 0.15, and no constraints. The XP score was employed to rank ligands and ascertain the best docked conformation for each ligand. The molecular mechanics–generalized Born surface area (MM-GBSA) binding free energy was computed for the top ranked poses using the Prime MM-GBSA module and set in context with the experimental binding affinities. Figures were produced with PyMol 3.1.3.1 Graphical Software (Schrödinger^®^, New York, NY, USA).

#### 3.3.8. Induced Fit Docking

Flexible docking was performed using the Induced-fit docking (IFD) tool in Maestro. Each ligand underwent an initial docking and then, side-chain prediction within 5 Å around every ligand pose [52]. This was accompanied by an energy minimization of each protein/ligand complex and a final prediction of the most favorable binding pose based on the IFD score [53].

## 4. Conclusions

A series of 11 new *N*-alkyl 3-(3-benzyloxyquinoxalin-2-yl) propanamides were prepared, based on the azide coupling of 3-(3-benzyloxyquinoxalin-2-yl) propanhydrazide with a variety of primary and secondary amines. This work targeted the chemoselective preparation of methyl 3-(3-benzyloxyquinoxalin-2-yl) propanoate and, in the best cases with higher yields, achieved this by the reaction of methyl 3-(3-oxo-3,4-dihydroquinoxalin-2-yl) propanoate with benzyl chloride in the presence of dry potassium carbonate in acetone under reflux condition for 12 h. The *O*-substituted benzyl derivative was hydrazinolyzed to provide the corresponding hydrazide. The compounds showed an interesting cytotoxic profile against a set of cancer cell lines including PC-3, Hela, HCT-116, and MCF-7. Compound **6k** had the highest activity an IC_50_ of values 12.17 ± 0.9, 9.46 ± 0.7, 10.88 ± 0.8, and 6.93 ± 0.4 µM compared to 8.87 ± 0.6, 5.57 ± 0.4, 5.23 ± 0.3, and 4.17 ± 0.2 µM for Doxorubicin against Hela, HCT-116, and MCF-7, respectively. The docking studies revealed the inhibition of HDAC-6 through binding the ubiquitin-binding domain and not the catalytic domain. This study produced a quinoxaline-based scaffold with a broad spectrum of antiproliferative activity and a unique mode of action through binding HDAC6 Zf-UBD that should be subjected to further optimization and preclinical testing and that can assist in future anticancer drug development.

## Figures and Tables

**Table 1:** Comparison of *O*- and *N*-substituted yields in different solvents.

Solvent	*O*-Substituted Yield	*N*-Substituted Yield
DMF	2%	85%
Acetonitrile	33%	55%
Ethanol	20%	38%
Acetone	63%	18%

**Table 2:** Cytotoxic activity of some compounds against tumor cell line.

Comp. No.	In Vitro Cytotoxicity IC_50_ (µM) *			
	PC-3	Hela	HCT-116	MCF-7
**2**	34.77 ± 2.2	18.16 ± 1.5	33.05 ± 2.1	24.83 ± 1.7
**3**	87.24 ± 4.5	59.33 ± 3.3	79.31 ± 3.9	61.71 ± 3.3
**4**	17.82 ± 1.3	13.44 ± 1.1	19.06 ± 1.3	11.19 ± 0.9
**6a**	94.14 ± 4.9	78.20 ± 3.8	81.65 ± 4.0	76.07 ± 3.9
**6b**	˃100	91.38 ± 4.8	˃100	85.20 ± 4.2
**6c**	65.06 ± 3.5	43.35 ± 2.6	49.31 ± 2.8	54.57 ± 3.1
**6d**	82.75 ± 4.1	69.48 ± 3.6	74.51 ± 3.7	72.64 ± 3.8
**6e**	˃100	63.59 ± 3.5	89.43 ± 4.5	67.72 ± 3.6
**6f**	47.61 ± 2.7	25.16 ± 1.7	38.76 ± 2.4	30.14 ± 1.9
**6g**	56.15 ± 3.2	32.21 ± 2.0	46.60 ± 2.7	37.54 ± 2.4
**6h**	77.83 ± 3.8	53.86 ± 3.1	60.63 ± 3.4	41.22 ± 2.6
**6i**	29.22 ± 1.8	15.50 ± 1.3	22.49 ± 1.6	17.37 ± 1.4
**6j**	70.19 ± 3.7	39.02 ± 2.5	55.71 ± 3.1	48.82 ± 2.8
**6k**	12.17 ± 0.9	9.46 ± 0.7	10.88 ± 0.8	6.93 ± 0.4
Doxorubicin	8.87 ± 0.6	5.57 ± 0.4	5.23 ± 0.3	4.17 ± 0.2

* IC_50_ (µM): 1–10 (very strong); 11–20 (strong); 21–50 (moderate); 51–100 (weak); above 100 (non-cytotoxic).

**Table 3:** Calculated XP scores, and experimental IC_50_ (µM) for compounds **2**, **3**, **4**, **6b**, **6g**, and **6k**.

Compound	XP Score (kcal/mol)	Rank	Experimental IC_50_-MCF-7 (µM)	Rank	Activity Threshold
**6k**	−6.11	1	6.93 ± 0.4	1	Active
**4**	−6.07	2	11.19 ± 0.9	2	Active
**2**	−5.27	3	24.83 ± 1.7	3	Moderate
**6g**	−4.93	5	37.54 ± 2.4	4	Moderate
**3**	−4.90	4	61.71 ± 3.3	5	Weak
**6b**	−4.63	6	85.20 ± 4.2	6	Inactive
Crystal ligand	−6.62	-	-	-	-

## Data Availability

Data are contained within the article.
